# Supplementary material for: Naphthalene-Based Oxime Esters as Type I Photoinitiators for Free Radical Photopolymerization
Source: Polymers (Basel). 2022 Dec 2;14(23):5261. doi: 10.3390/polym14235261 (PMC9735988; doi:10.3390/polym14235261)
Supplement: Supplementary file 1 [file polymers-14-05261-s001.zip › polymers-2041721-supplementary.pdf]

Supporting Information

Naphthalene-based oxime esters as Type I photoinitiators for free radical photopolymerization

Zhong-Han Lee[a], Fatima Hammoud[b,c,d], Tung-Liang Huang[a], Akram Hijazi[d], Bernadette Graff[b,c], Jacques Lalevée[b,c*], Yung-Chung Chen[a,e*]

[a.] Department of Chemical and Materials Engineering, National Kaohsiung University of Science and Technology, Kaohsiung 80778, Taiwan

[b.] Université de Haute-Alsace, CNRS, IS2M UMR 7361, F-68100, Mulhouse, France

[c.] Université de Strasbourg, France

[d.] EDST, Université Libanaise, Campus Hariri, Hadath, Beyrouth, Liban

[e.] Photo-SMART (Photo-sensitive Material Advanced Research and Technology Center), National Kaohsiung University of Science and Technology, Kaohsiung City 80778, Taiwan

*Corresponding authors: JacquesLalevée (jacques.lalevee@uha.fr)

Yung-Chung Chen (chenyc@nkust.edu.tw)

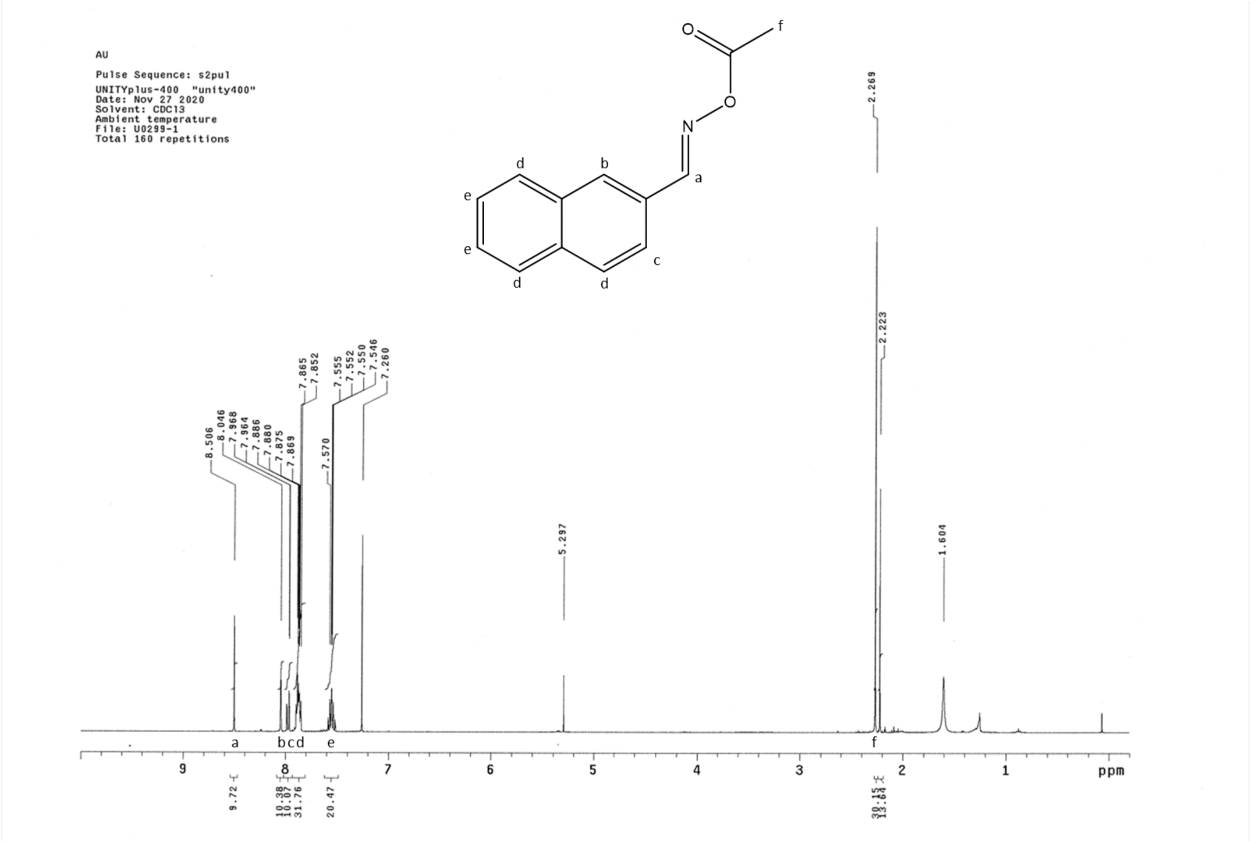

Figure S1 ^{1}H NMR of the **NA-1**.

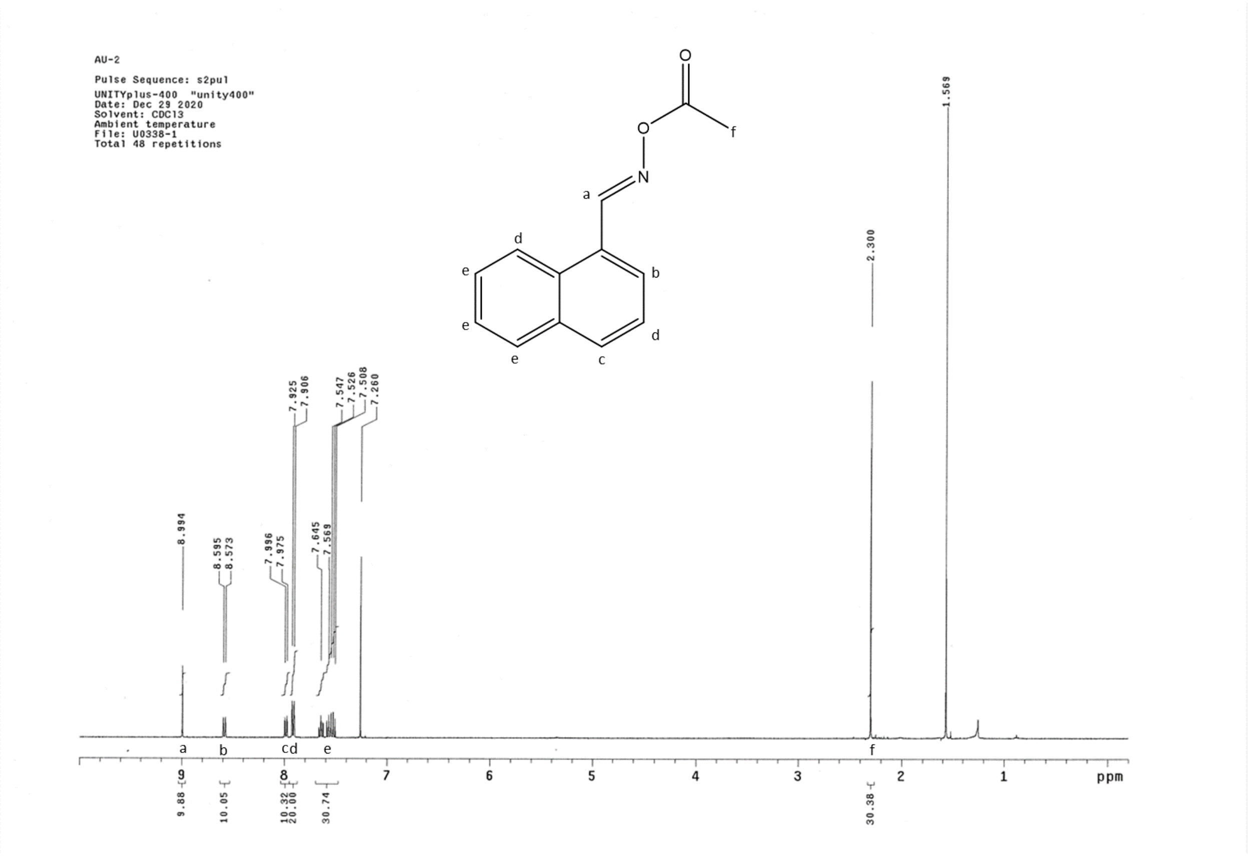

Figure S2 ^{1}H NMR of the **NA-2**.

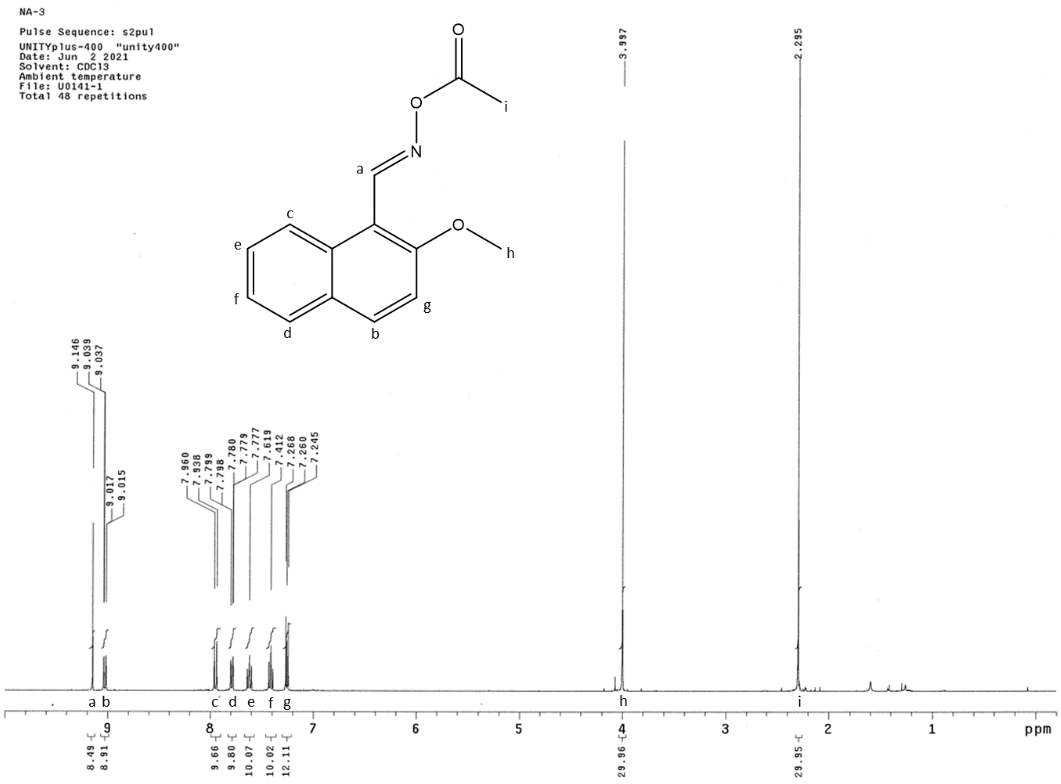

Figure S3 ^{1}H NMR of the **NA-3**.

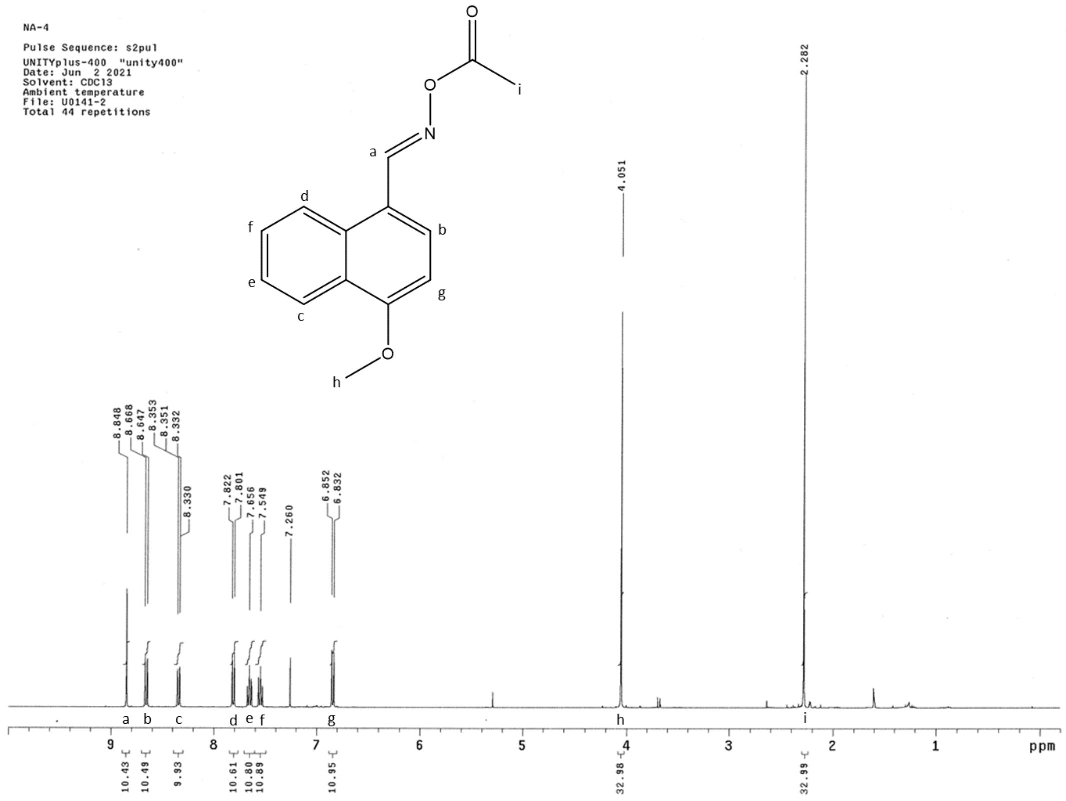

Figure S4 ^{1}H NMR of the **NA-4**.

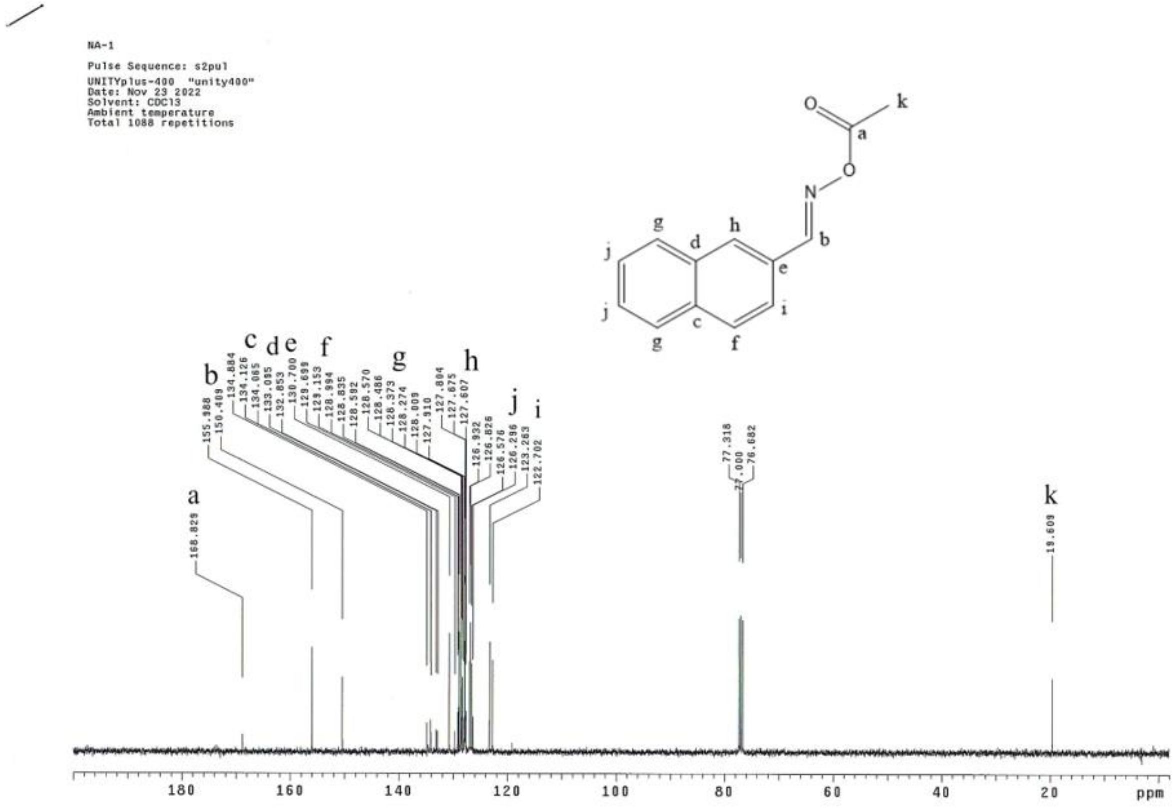

Figure S5 ^{13}C NMR of the **NA-1**.

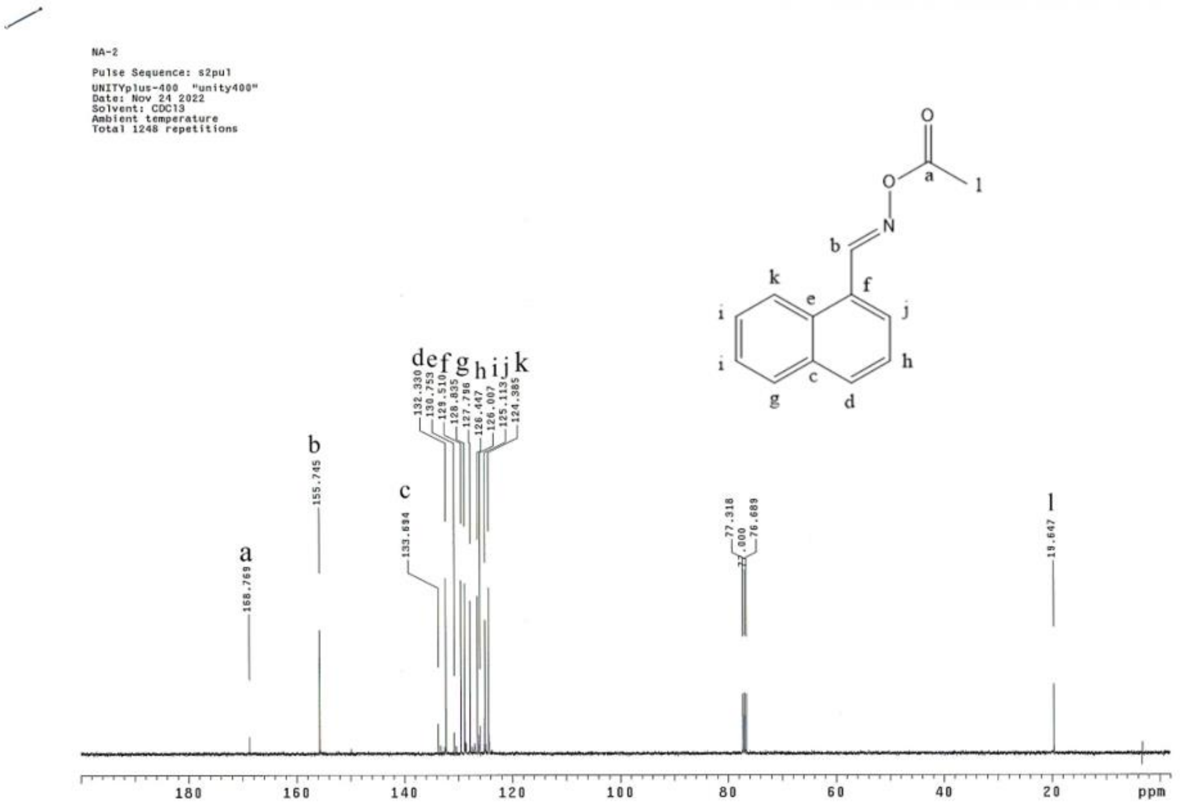

Figure S6 ^{13}C NMR of the **NA-2**.

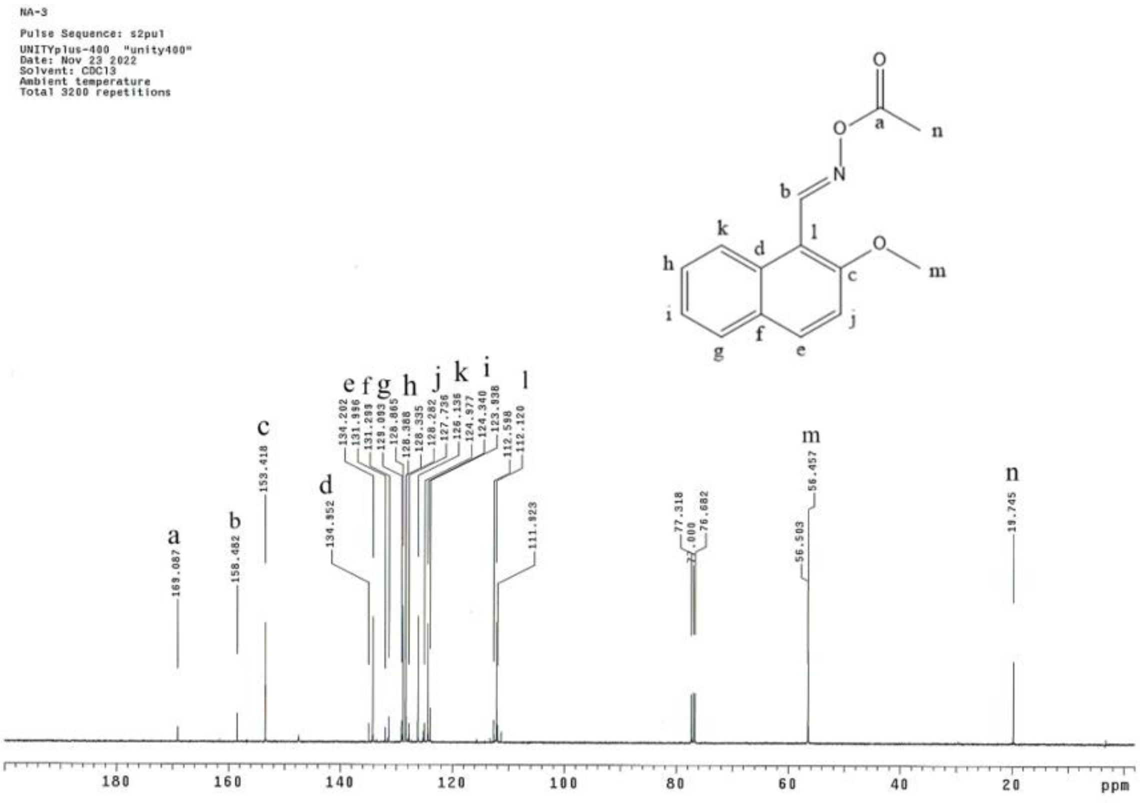

Figure S7 ^{13}C NMR of the **NA-3**.

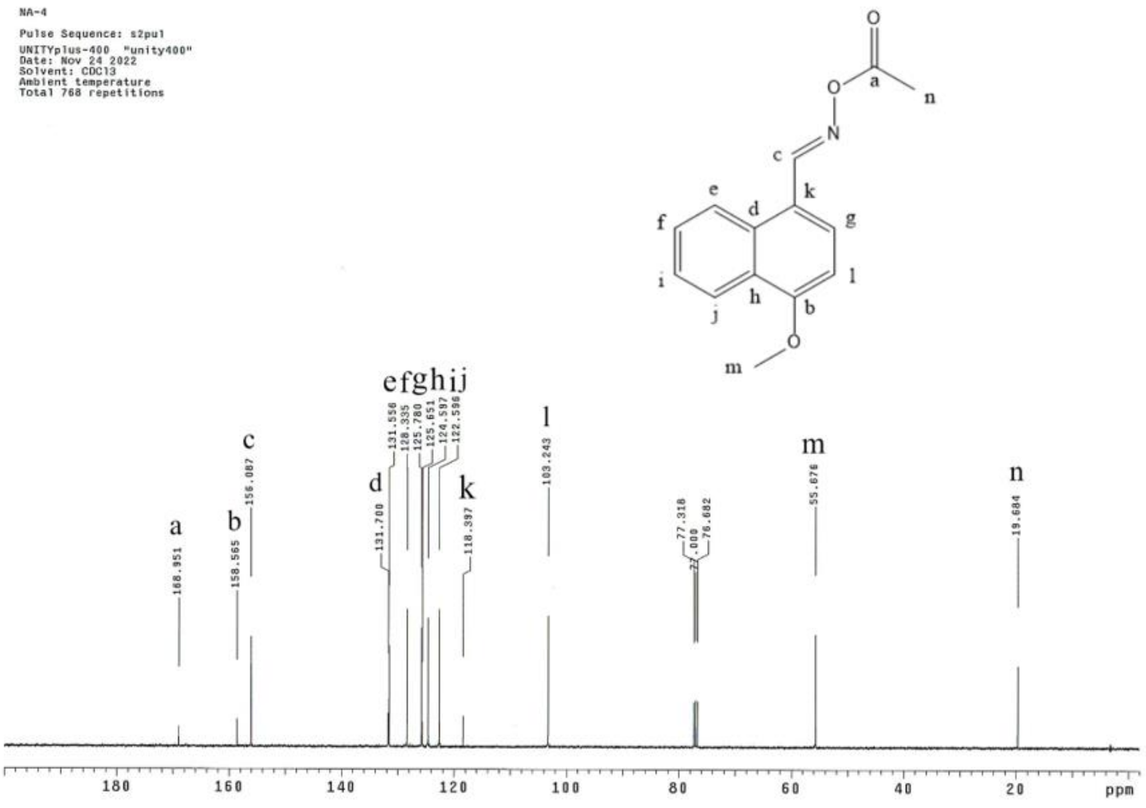

Figure S8 ^{13}C NMR of the **NA-4**.

FT-MS

Analysis Info

Analysis Name	D:\Data\g8\AU_000001.d		3/11/2021 11:55:02 AM
Method	broadband first signal		
Sample Name	AU		Instrument: FT-MS solariX
Comment	ESI Positive		

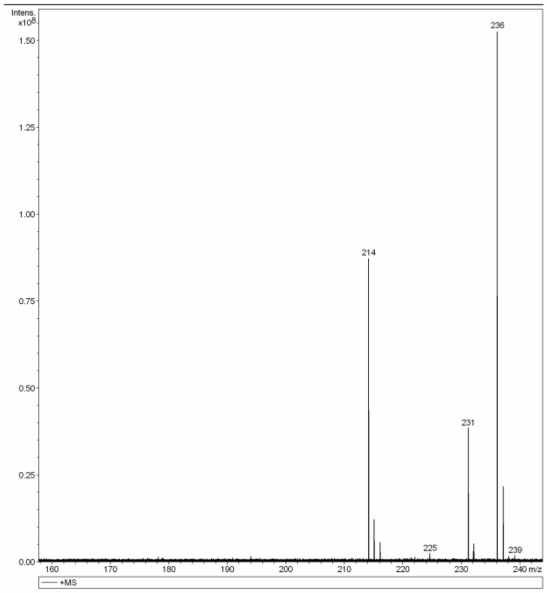

Bruker Compass DataAnalysis 4.0 printed: 3/11/2021 11:55:02 AM Page 1 of 1

Mass Spectrum SmartFormula Report

Analysis Info

Analysis Name	D:\Data\g8\AU_000002.d	3/11/2021 11:54:20 AM
Method	broadband first signal	Operator: YU HSIAO-CHING
Sample Name	AU	Instrument: BRUKER FT-MS solariX
Comment	ESI Positive	

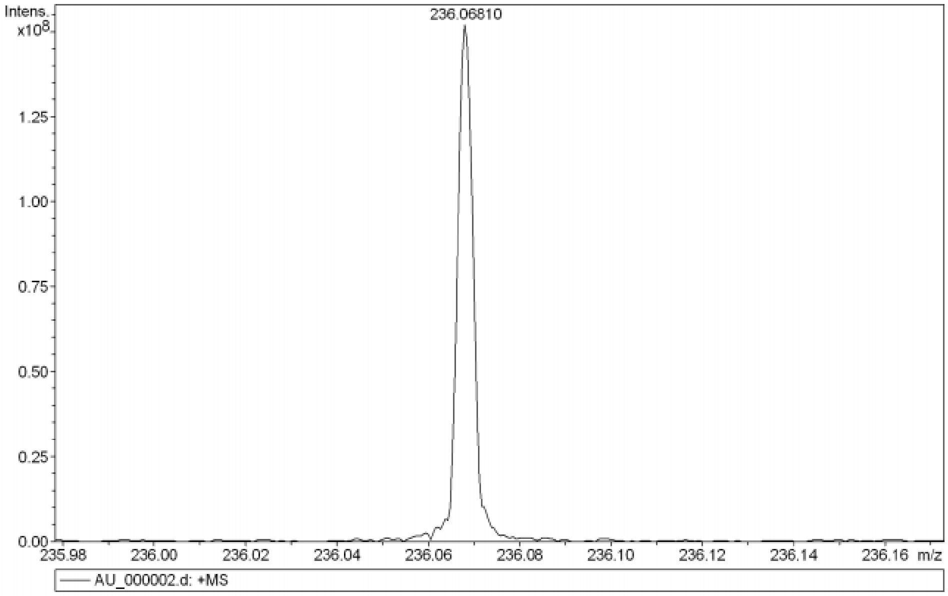

Meas. m/z	#	Formula	Score	m/z	err [mDa]	err [ppm]	mSigma	rdb	e⁻ Conf	N-Rule
236.06810	1	C 13 H 11 N Na O 2	100.00	236.06820	0.10	0.42	6.8	8.5	even	ok

Figure S9 Fourier-transfer mass spectrometry (FT-MS) of the **NA-1**.

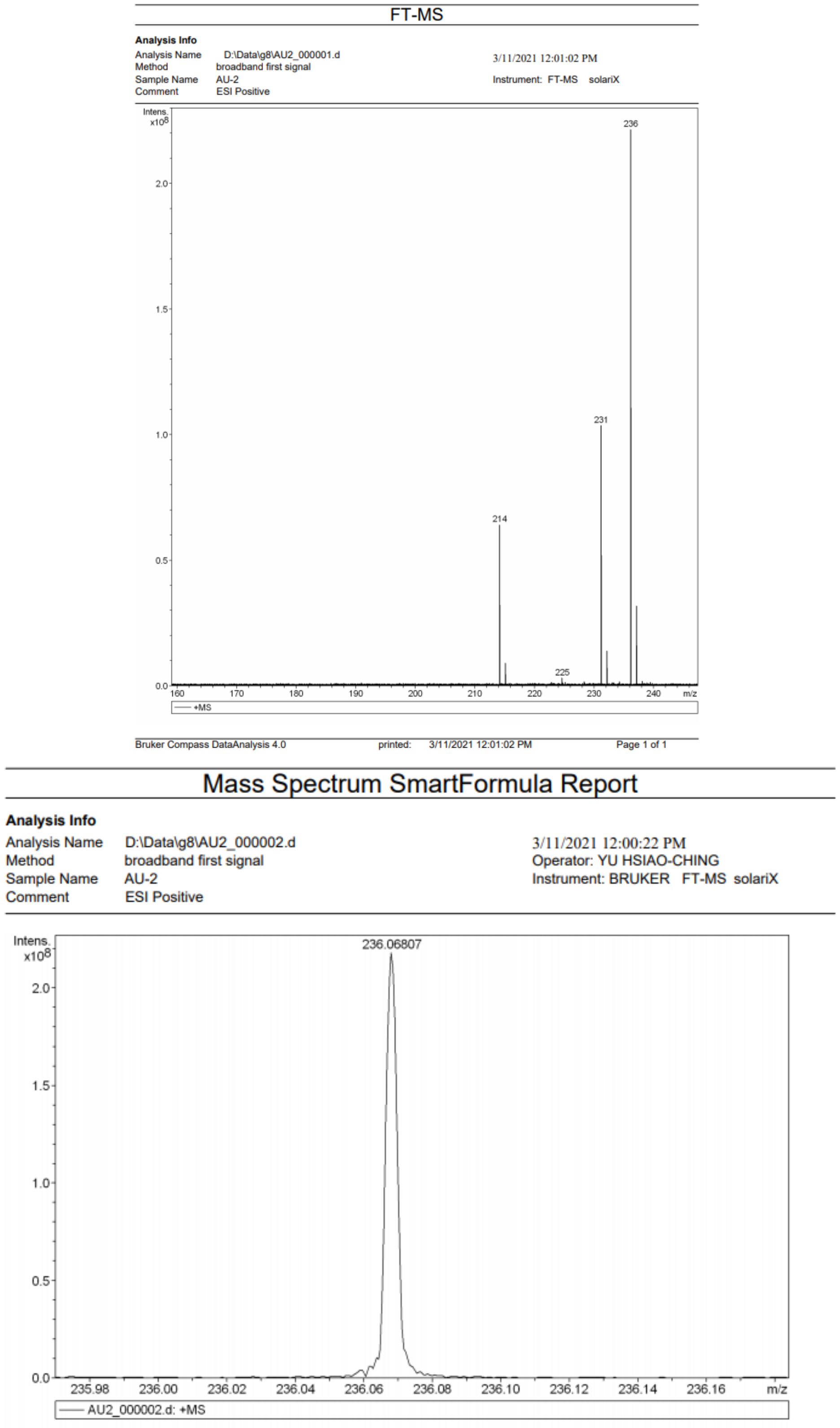

Meas. m/z	#	Formula	Score	m/z	err [mDa]	err [ppm]	mSigma	rdb	e¯ Conf	N-Rule
236.06807	1	C 13 H 11 N Na O 2	100.00	236.06820	0.13	0.55	2.0	8.5	even	ok

Figure S10 Fourier-transfer mass spectrometry (FT-MS) of the **NA-2**.

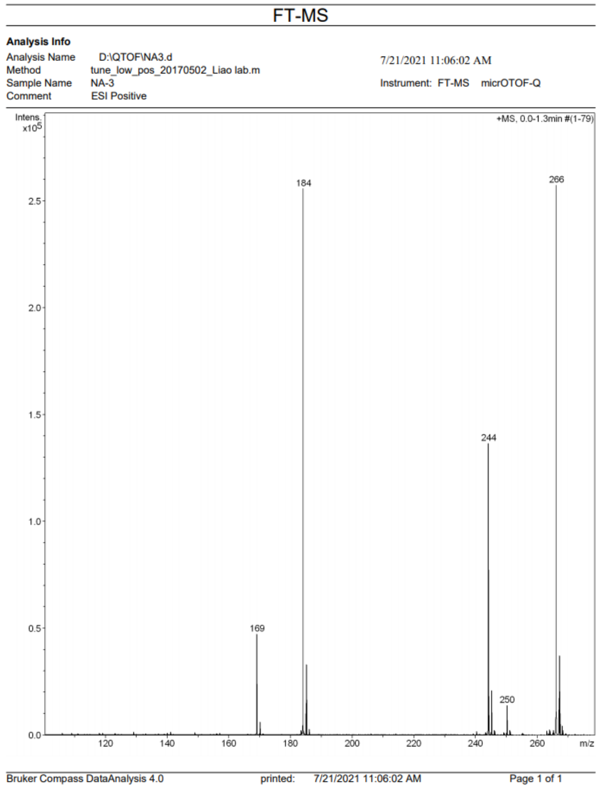

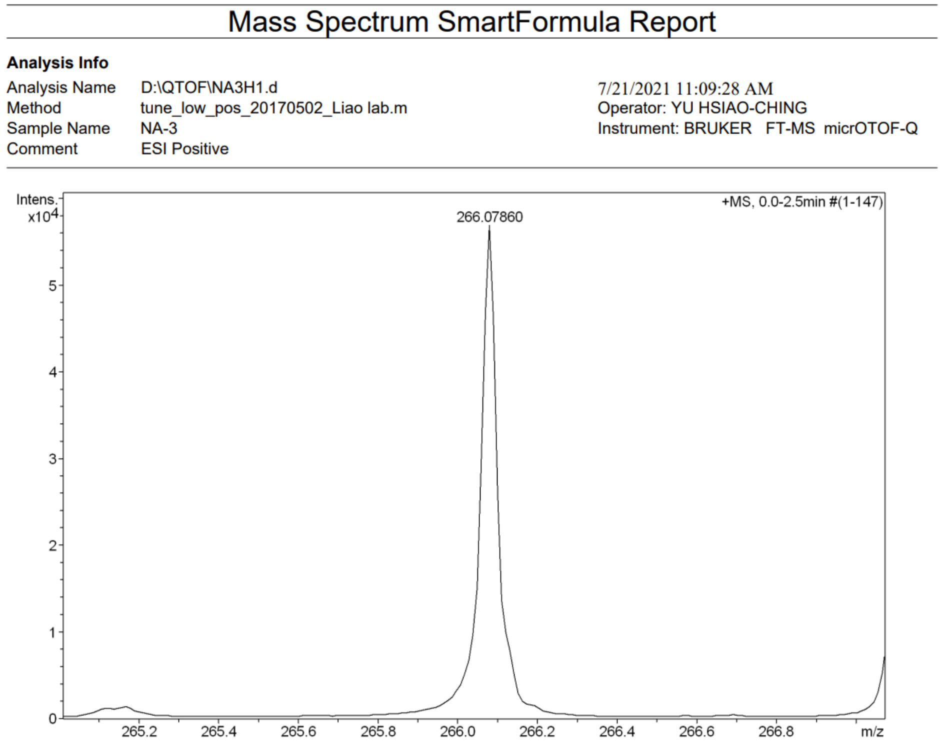

Meas. m/z	#	Formula	Score	m/z	err [mDa]	err [ppm]	mSigma	rdb	e⁻ Conf	N-Rule
266.07860	1	C 14 H 13 N Na O 3	100.00	266.07876	0.17	0.63	2.3	8.5	even	ok

Figure S11 Fourier-transfer mass spectrometry (FT-MS) of the **NA-3**.

FT-MS

Analysis Info

Analysis Name D:\QTOF\NA4.d
Method tune_low_pos_20170502_Liao lab.m
Sample Name NA-4
Comment ESI Positive

7/21/2021 11:10:39 AM
Instrument: FT-MS micrOTOF-Q

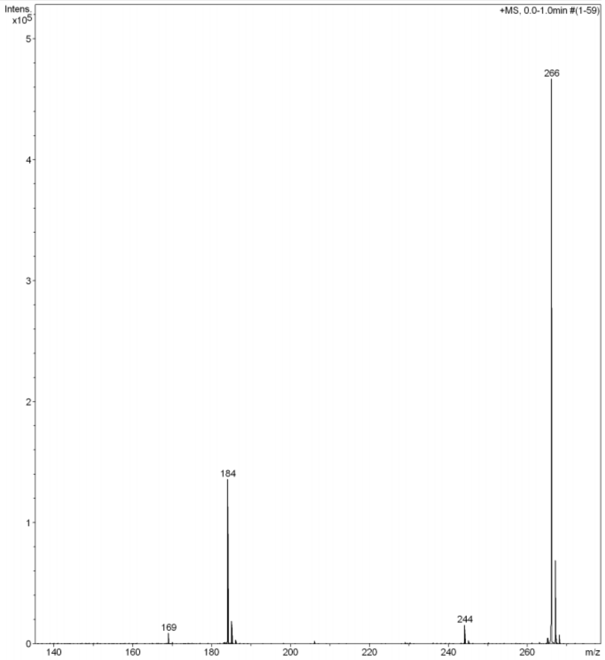

Bruker Compass DataAnalysis 4.0 printed: 7/21/2021 11:10:39 AM Page 1 of 1

Mass Spectrum SmartFormula Report

Analysis Info

Analysis Name D:\QTOF\NA4H3.d
Method tune_low_pos_20170502_Liao lab.m
Sample Name NA-4
Comment ESI Positive

7/21/2021 11:12:29 AM
Operator: YU HSIAO-CHING
Instrument: BRUKER FT-MS micrOTOF-Q

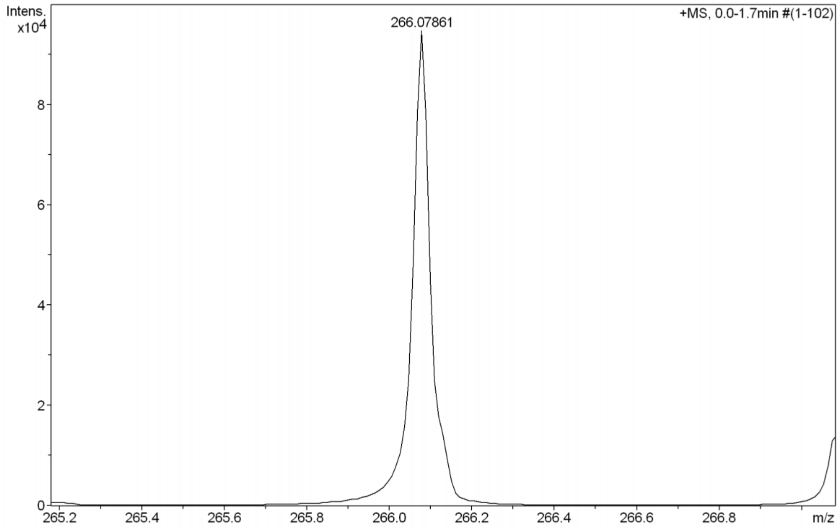

Meas. m/z	#	Formula	Score	m/z	err [mDa]	err [ppm]	mSigma	rdb	e⁻ Conf	N-Rule
266.07861	1	C 14 H 13 N Na O 3	100.00	266.07876	0.16	0.59	4.4	8.5	even	ok

Figure S12 Fourier-transfer mass spectrometry (FT-MS) of the **NA-4**.
